# Herpes simplex virus type 1 interferes with signal transducer and activator of transcription 3 expression in infected mature dendritic cells

**DOI:** 10.1099/jgv.0.002280

**Published:** 2026-08-12

**Authors:** Alexandra Birzer, Christina Drassner, Petra Muehl-Zuerbes, Christine Kuhnt, Adalbert Krawczyk, Andrea K. Thoma-Kress, Alexander Steinkasserer, Linda Popella

**Affiliations:** 1 Harald zur Hausen Institute of Virology, Uniklinikum Erlangen, Friedrich-Alexander-Universität Erlangen-Nürnberg (FAU), Erlangen, Germany; 2 Department of Immune Modulation, Uniklinikum Erlangen, Friedrich-Alexander-Universität Erlangen-Nürnberg (FAU), Erlangen, Germany; 3 Department of Infectious Diseases, West German Centre of Infectious Diseases, University Medicine Essen, University Hospital Essen, University Duisburg-Essen, Essen, Germany; 4 Institute for Virology, University Hospital Essen, University Duisburg-Essen, Essen, Germany; 5 FAU Profile Center Immunomedicine (FAU I-MED), Friedrich-Alexander-Universität Erlangen-Nürnberg (FAU), Erlangen, Germany; 6 Institute of Molecular Infection Biology (IMIB), University of Würzburg, Würzburg, Germany

**Keywords:** dendritic cell, herpes simplex virus type 1 (HSV-1), IL-6 signalling pathway, immune modulation, signal transducer and activator of transcription 3 (STAT3)

## Abstract

Herpes simplex virus type 1 (HSV-1) is the prototype of the *α*-herpesvirus family. To propagate within the host organism, HSV-1 has evolved several strategies to subvert the host immune response. Due to the pivotal role of dendritic cells (DCs), bridging innate and adaptive immunity and activating naïve T cells, they represent an attractive target for HSV-1-triggered immune regulation. Here, we report a novel HSV-1-mediated mechanism of signal transducer and activator of transcription 3 (STAT3) dysregulation in human monocyte-derived mature DCs (mDCs). Due to STAT3’s antiviral activity, STAT3 was shown to be modulated by viruses to efficiently replicate in several different cell types, however, not in DCs. We show that HSV-1 infection of mDCs leads to diminished total STAT3 expression and STAT3 phosphorylation (pSTAT3), as well as downregulation of *STAT3* mRNA very early upon infection. Protein downregulation of STAT3 could be verified by label-free mass spectrometric analysis of HSV-1- and HSV-2-infected mDCs. We found that two viral proteins, the tegument protein virion host shutoff (vhs) and the immediate-early protein ICP27, contribute to STAT3 protein downregulation upon HSV-1 infection. However, HSV-1 Δvhs and ΔICP27 deletion strains still inhibited pSTAT3 in infected mDCs, suggesting that different viral proteins impair STAT3 phosphorylation and total STAT3. Moreover, STAT3 protein levels were degraded in a proteasome-dependent but apoptosis-independent manner. Taken together, we demonstrate that HSV-1 downregulates STAT3 in HSV-1-infected human mDCs at the transcript, protein and phosphorylation levels. Downregulation of STAT3 protein depended on vhs and the proteasome, very likely to shape DC function for immune evasion and modulation.

**Impact Statement**

By engulfing and presenting foreign antigens, dendritic cells (DCs) link the innate and adaptive immunity. Antigen presentation of DCs to naïve T cells is essential to provoke an effective immune response. Thus, this unique cell type is often functionally and phenotypically dysregulated by pathogens, such as viruses. In this study, we identified the cellular signal transducer and activator of transcription 3 (STAT3) protein to be downregulated in human mature DCs by the *α*-herpesvirus herpes simplex virus type 1 (HSV-1). We identified contributing viral proteins and the mechanism to be responsible for the observed dysregulation of STAT3. Since STAT3 is described to act antiviral, HSV-1 might degrade this cellular protein to circumvent STAT3’s antiviral activity upon infection.

## Introduction

Herpes simplex virus type 1 (HSV-1) belongs to the *α*-herpesvirus family and represents the prototype of this subfamily. A characteristic of the *α*-herpesvirus family is the infection of a wide range of host species including infection of different cell types, e.g. epithelial cells, fibroblasts and immune cells, such as dendritic cells (DCs) [1–3]. Due to its high seroprevalence in Germany [4], ranging from ~75 to 80%, HSV-1 is a widely distributed and clinically relevant pathogen, remaining either asymptomatic or causing vesicular lesions (*Herpes labialis*) in immunocompetent patients [5]. These common lesions represent the viral reservoir for further intra- or interhost virus transmission. However, in case of immunosuppression or reduced immunocompetency, the reactivation and replication of HSV-1 leads to severe keratitis and encephalitis in corneal epithelial cells or in the brain, respectively [6–8]. Upon reactivation, transcription of the 150 kb linear double-stranded HSV-1 DNA genome is initiated in a tripartite gene expression cascade [9]. The immediate-early (IE) phase is followed by transcription of the early (E) proteins for subsequent activation of transcription and translation of structural late (L) proteins [10]. Thus, lytic infection will give rise to infectious viral particles, composed of DNA protected within the capsid, the tegument layer and an outer envelope.

For efficient viral replication, HSV-1 has evolved different strategies to interfere with the host’s immune response and to evade antiviral mechanisms. DCs are crucial immune cells that link innate and adaptive immunity. They play a pivotal role in the establishment of an effective immune response due to their capacity to activate naïve T cells. To do so, sessile immature DCs (iDCs) scan for foreign peptides, such as those present on pathogens, for their subsequent capturing and intracellular processing. This is accompanied by a maturation process that is characterized by the upregulation of chemokine receptors, e.g. CC chemokine receptor (CCR) 7 and CXC chemokine receptor 4 and surface receptors essential for T cell stimulation, such as cluster of differentiation (CD) 40, CD80, CD86 and CD83. By fundamental changes in the expression pattern upon maturation, mature DCs (mDCs) are able to present antigens on their cell surface via major histocompatibility complex (MHC) class I and II molecules in the lymph node to naïve T cells, triggering the induction of an adaptive immune response. Based on these unique and vital functions between innate and adaptive immunity, DCs are often targeted by viruses for immune evasion [11–13]. Dependent on the virus type, DCs are differentially modified in their function. Specifically, HSV-1 interferes with DC migration by inhibition of CCR7 surface expression, which consequently reduces the chemokine sensitivity [14]. Another important example of HSV-1-mediated interference with DC function is the degradation of the functionally important CD83 molecule. Due to the loss of CD83, peptide presentation is impaired, and HSV-1-infected mDCs have reduced T cell stimulatory capacity [15, 16].

The signal transducer and activator of transcription 3 (STAT3) is a functionally important signalling transcription factor that is stimulated by cytokine receptor binding, such as IL-6 to IL-6 receptor (IL-6R) or IL-11 to IL-11 receptor with its co-receptor gp130, which is ubiquitously expressed on all cell types [17–19]. The co-receptor gp130 is associated with the Janus kinase (JAK) [20–22], which is subsequently activated by auto-phosphorylation and transfers the phosphate group to the intracellular domains of gp130 [23, 24]. STAT3 is functionally inactive in the cytoplasm, and after the recruitment of cytoplasmic STAT3 to phosphorylated gp130, STAT3 itself is phosphorylated by JAKs and translocates as a protein dimer into the nucleus. STAT3 interacts with DNA via its DNA-binding domain and activates gene expression of several downstream targets, such as IL-6 responsive genes, for the regulation of proliferation, survival, differentiation and apoptosis [25–27].

Moreover, STAT3 expression is important for DC function and T cell immunity [28, 29]. In DCs, STAT3 is functionally important to balance activation and tolerance. Depletion of STAT3 in knock-out mice induced cervical lymphadenopathy and ileocolitis, while the specific deletion of STAT3 in DCs fosters immune activity [30]. Besides this, STAT3 is a common and frequent target for modulation by herpesviruses such as Varicella Zoster virus (VZV), human cytomegalovirus (HCMV) or Kaposi’s sarcoma-associated herpesvirus (KSHV) [31]. Viral manipulation of STAT3 substantially varies between different viruses and especially between different herpesviruses, but also between cell types [31, 32]. Since mice were more susceptible to HSV-1 in the absence of STAT3, STAT3 has also been proposed to have antiviral properties towards HSV-1 in murine cells [33]. Besides this, DCs function as regulators of HSV-1 latency in the trigeminal ganglia [34], and STAT3 was demonstrated to play an essential role in HSV-1 reactivation [35].

Here, we report a novel mechanism of HSV-1-mediated STAT3 dysregulation in primary human immune cells, specifically monocyte-derived mDCs. HSV-1 selectively degrades STAT3 in human mDCs at both the protein and mRNA levels. We identified the viral tegument protein vhs and the IE protein ICP27 contributing to STAT3 downregulation together with the cellular proteasome machinery. Overall, our data suggest that HSV-1 downregulates STAT3 in mature DCs to interfere with its antiviral activity to foster viral propagation.

## Methods

### Virus amplification

The herein designated HSV-1 wt strain originates from the laboratory strain HSV-1 syn 17^+^ [36] and has been modified by insertion of a GFP cassette into the *UL43* locus of the HSV-1 genome (BioVex): HSV-1/syn 17^+^/CMV-EGFP/UL43 [cytomegalovirus (CMV), EGFP and unique long (UL)]. GFP expression is regulated by the *CMV* promoter, and the deletion of the viral *UL43* gene has been described to be non-essential for HSV-1 replication [37, 38]. For HSV-2 infection, we used the HSV-2 strain G (provided by A. Krawczyk).

The following deletion strains, generated from the HSV-1 syn 17^+^ laboratory strain, were used: HSV-1 pR20.5/vhs (HSV1- Δvhs), HSV-1 ∆ICP0/YFP-ICP4 (HSV-1 ΔICP0) and HSV-1 27-pR19 lacZ (HSV-1 ΔICP27). The HSV-1 talimogene laherparepvec (T-VEC) originates from the pharmaceutical drug T-VEC (IMLYGIC®), used as an HSV-1 ΔICP34.5/ΔICP47 strain [39]. The HSV-1 Δvhs strain has an additional *EGFP-lacZ* gene cassette inserted into the *UL41* gene, which encodes the viral vhs (kindly provided by Martin Messerle, Hannover Medical School, Germany). The viral ICP27 protein is ablated in HSV-1 ΔICP27 by the insertion of a *lacZ* gene cassette into this gene. The clinical isolates are viruses isolated from patients with acute HSV-1 infection and were provided by Manfred Marschall, Uniklinikum Erlangen.

The permissive cell line baby hamster kidney (BHK) 21 was used for amplification of HSV-1 wt, HSV-2, HSV-1 Δvhs and HSV-1 clinical isolates. The deletion strain HSV-1 ΔICP27 was propagated on BHK M49 cells and HSV-1 T-VEC on Vero cells. HSV-1 ∆ICP0/YFP-ICP4 was amplified on the U2OS cell line. In brief, 15 T_175_ cell culture flasks containing the respective cell line were washed once with PBS and infected with infection medium [Roswell Park Memorial Institute (RPMI) 1640 (Lonza, Switzerland), 20 mM HEPES], supplemented with the respective HSV-1 strain at an multiplicity of infection (MOI) of 0.01. After 1 h of infection at 24 °C, 20 ml Dulbecco’s modified Eagle’s medium [supplemented with 10% FCS, 2 mM l-glutamine, 100 U ml^−1^ penicillin, 100 U ml^−1^ streptomycin and 1% non-essential amino acids (100 × stock)] were added per cell culture flask. Cells were incubated at 37 °C and 5% CO_2_ for 4 days. Subsequently, the HSV-1 particle containing supernatants were centrifuged at 2,575 g at 4 °C for 10 min to remove cell debris. The viral particles were isolated by high-speed centrifugation at 39,742 g at 4 °C for 2 h. Finally, 150 μl buffer containing 30 mM MES, 100 mM NaCl and 20 mM Tris was added to each cell pellet, and the samples were stored at 4 °C overnight. The virus suspension was aliquoted and stored in cryo-vials at −80 °C. Virus titration was performed as previously described [40].

### Human monocyte-derived DC generation

The isolation of peripheral blood mononuclear cells (PBMCS) was previously published [41, 42]. First, blood from the leukoreduction system chamber (LRSC) was diluted into PBS containing 10% anticoagulant citrate dextrose solution, solution A (Lonza) and pipetted onto the lymphoprep solution (Nycomed Pharma AS, Norway). The gradient was centrifuged at 400 ***g*** at 24 °C for 30 min. The mononuclear cells form a distinct band, which was collected and washed three times with ice-cold PBS containing 1 mM EDTA, washed once in RPMI 1640 (Lonza) via centrifugation at 300 ***g*** for 5 min and resuspended in 25 ml DC medium (RPMI 1640 supplemented with 1% human AB serum (Sigma-Aldrich, Germany), 100 U ml^−1^ penicillin and 100 U ml^−1^ streptomycin, 2 mM l-glutamine and 10 mM HEPES (all Lonza). Monocytes were adhered to the bottom for 1 h at 37 °C and 5% CO_2_. The non-adherent fraction was removed, and adherent cells were washed three times with RPMI 1640. After a second time of adherence, monocytes were cultivated in 30 ml of DC medium supplemented with 800 U ml^−1^ granulocyte-macrophage colony-stimulating factor (GM-CSF) (Miltenyi Biotec, Germany) and 250 U ml^−1^ IL-4 (Miltenyi Biotec) for DC differentiation. On day four after adherence, cells were fed with 5 ml fresh DC medium containing 400 U ml^−1^ GM-CSF and 250 U ml^−1^ IL-4. On the following day, iDCs in each T_175_ cell culture flask were matured by the addition of a maturation cocktail composed of GM-CSF (40 U ml^−1^), IL-4 (250 U ml^−1^), IL-1*β* (Cell Genix GmbH, Germany; 200 U ml^−1^), IL-6 (Cell Genix GmbH; 1,000 U ml^−1^), TNF-*α* (Peprotech, Germany; 10 ng ml^−1^) and prostaglandin E2 (Pfizer, Germany; 1 µg ml^−1^). Two days post-maturation, mDCs were used for experiments and maturation was controlled by analyses of surface receptor expression by flow cytometry as described in [43].

### HSV-1 and HSV-2 infection of mDCs

HSV-1 and HSV-2 infection was performed with a defined cell number of mDCs (around 1 to 4×10^6^ mDCs). To adjust the indicated MOI cells were mock-, HSV-1- or HSV-2-infected in 300 µl infection medium supplemented with the respective amount of the respective HSV stock into 2 ml tubes. After an infection time of 1 h at 37 °C and 350 r.p.m., cells were separated from the infection medium by centrifugation at 3,390 ***g*** for 2 min. Subsequently, cells were resuspended in DC medium (containing 40 U ml^−1^ GM-CSF and 250 U ml^−1^ IL-4) at a final concentration of 1×10^6^ mDCs/ml and incubated in well plates at 37 °C and 5% CO_2_.

### Inhibitor treatment

The effect of IE proteins on STAT3 expression levels was analysed by performing cycloheximide (CHX)/actinomycin D (Act. D) chase experiments. In brief, mDCs were mock- or HSV-1 wt-infected (MOI of 2) in the presence of the translation inhibitor CHX (100 µg ml^−1^, Sigma-Aldrich) or DMSO as a control. At 1 h post-infection (hpi), cells were centrifuged at 3,390 ***g*** for 2 min to remove infectious medium and subsequently resuspended in fresh DC medium supplemented with 40 U ml^−1^ GM-CSF, 250 U ml^−1^ IL-4 and 100 µg ml^−1^ CHX or Dimethyl sulfoxide (DMSO) as a control and seeded into well plates. Later (5 hpi), cells were washed twice with RPMI 1640 either supplemented with the transcription inhibitor Act. D (5 µg ml^−1^, Sigma-Aldrich) or DMSO as a control. Afterwards, cells were seeded into well plates and incubated with fresh DC medium supplemented with 40 U ml^−1^ GM-CSF, 250 U ml^−1^ IL-4 and 5 µg ml^−1^ Act. D or DMSO until cells were harvested at 20 hpi.

To analyse whether the proteasome, autophagy or lysosomal degradation is involved in protein degradation, the inhibitors MG-132 (10 µM, Enzo Life Science, Germany), bortezomib (BZ, 2 µM, Santa Cruz Biotechnology, Germany) or lactacystin D (LacD, 10 µM, Biomol, Germany) were added at 1 hpi into the DC medium. The inhibitors chloroquine (50 µM, Enzo Life Science) or bafilomycin A1 (BA-1, 500 nM, Sigma-Aldrich) were applied 1 h prior to infection. To block apoptosis, mDCs were mock- or HSV-1 wt-infected in the presence of the pan-caspase inhibitor Z-VAD-FMK (zVAD, 20 µM; InvivoGen, USA) or DMSO as a control. The inhibitor or DMSO was added additionally to the DC medium at 1 hpi.

### Preparation of protein lysates and Western blotting

For protein lysis, cells were harvested and washed once with ice-cold PBS. Pellets were either stored at −80 °C or directly lysed for 20 min on ice in 35 µl of sodium deoxycholate lysis buffer (10% glycerol, 2 mM EDTA, 137 mM NaCl, 50 mM Tris pH 8.0 and 0.5% deoxycholate), which was freshly supplemented with 2 mM phenylmethylsulfonyl fluoride, 2 mM sodium orthovanadate, 20 mM sodium fluoride, 10 mM MgCl_2_ and benzonase. Cell lysates were spun down at 15,900 ***g*** at 4 °C for 20 min. The protein concentration of the supernatants was subsequently determined using Bradford protein determination reagents (Roti Quant, Carl Roth GmbH, Germany). Finally, protein lysates were adjusted to a defined protein concentration using H_2_O and 4× Roti-Load 1 (final concentration: 1×; Carl Roth GmbH) and boiled at 95 °C for 10 min. Alternatively, cell pellets were resuspended in Roti-Load lysis mix composed of 4× Roti-Load 1 (final concentration: 2×), 12.5 U ml^−1^ benzonase and 1 mM MgCl_2_ and lysed 10 min at 37 °C, and finally, lysates were boiled at 95 °C for 10 min.

For Western blot analyses, cell lysates were loaded onto 10% SDS polyacrylamide gels and subsequently separated using SDS-PAGE. By semi-dry Western blot transfer, proteins were transferred onto a nitrocellulose membrane (Amersham, GE Healthcare, Germany). The membrane was blocked for 1 h in 1× Roti-block (Carl Roth GmbH). Afterwards, the membrane was incubated with the primary antibodies and diluted in 1× Roti-block, overnight at 4 °C. The Western blot was washed five times and incubated with HRP-conjugated secondary antibodies, membranes were washed again five times and proteins were detected using Amersham ECL Prime Western blotting detection reagent (GE Healthcare) via the Image Quant LAS 4000 (GE Healthcare). All primary antibodies were diluted in 1× Roti-block and used as follows: mouse-anti-glycoprotein B (gB) antibody (Santa Cruz Biotechnology, sc-56987, clone 10B7, 1:1,000), mouse-anti-ICP4 antibody (Santa Cruz Biotechnology, sc-56986, clone 10F1, 1:1,000), mouse-anti-ICP0 antibody (Santa Cruz Biotechnology, sc-53070, clone 11060, 1:1,000), mouse-anti-glyceraldehyde-3-phosphate dehydrogenase (GAPDH) antibody (EMD Millipore Corp., clone MAB374, 1:5,000), mouse-anti-gD antibody (Santa Cruz Biotechnology, sc-69802, clone H170, 1:1,000), mouse-anti-ICP27 antibody (Santa Cruz Biotechnology, sc-69807, clone H1113, 1:1,000), mouse-anti-ICP8 antibody (Santa Cruz Biotechnology, sc-53329, clone 10A3, 1:1,000), rabbit-anti-STAT1 antibody (Cell Signaling Technology, #9172, 1:1,000), rabbit-anti-pSTAT3 antibody (Cell Signaling Technology, #9145, clone D3A7, 1:2,000, tyrosine 705) and mouse-anti-STAT3 antibody (Santa Cruz Biotechnology, Sc-8019, clone F-2). Secondary antibodies were diluted in 1× TBS-T: polyclonal anti-mouse-IgG HRP-linked (Cell Signaling Technology, #7076, 1:2,500) or polyclonal anti-rabbit IgG HRP-linked (Cell Signaling Technology, #7074, 1:2,000). Densiometric quantification of the protein expression was performed by using AIDA Image Analyzer v4.23 (Raytest Isotopenmessgeräte GmbH, Straubenhardt, Germany).

### Mass spectrometry (MS)

Protein samples of mock, HSV-1- (MOI of 2) and HSV-2-infected (MOI of 5) mDCs were prepared at 8 and 16 hpi. Cell pellets were lysed for 20 min on ice in 50 µl of sodium deoxycholate lysis buffer (10% glycerol, 2 mM EDTA, 137 mM NaCl, 50 mM Tris pH 8.0 and 0.5% deoxycholate), which was freshly supplemented with 2 mM phenylmethylsulfonyl fluoride, 2 mM sodium orthovanadate, 20 mM sodium fluoride, 10 mM MgCl_2_ and benzonase. Afterwards, cell lysates were incubated for 10 min at 37 °C, followed by centrifugation at 15,900 ***g*** for 15 min at 4 °C. The protein concentration was determined using Bradford protein determination reagents (Roti Quant, Carl Roth GmbH). Approximately 2 µg of the proteins was used for sample preparation as already described in Birzer *et al*.[44]. Briefly, samples were prepared according to the filter-aided sample preparation method [45]. Peptides were isolated using 10 kD cutoff filter (Microcon YM-10, Vivacon 500; Sartorius). Approximately 10 µg of each peptide solution was used for analysis in reverse phase chromatography with a linear increase of acetonitrile on a nano flow UltiMate 3000 HPLC (Dionex) with a flow rate of 200 nl min^−1^. By ionization of separated peptides by EASY-Spray ion source (Thermo Fisher Scientific, 2.0 kV, 275 °C), samples were analysed by an Orbitrap Fusion tribrid (Thermo Scientific) working in a positive polarity mode, and the peak areas of the peptides were integrated over time. Details on this method are shown in a previous publication [46]. For evaluation, the intensity (‘sum of the ion peak intensities of unique peptides’) of the detected peptide is normalized to the respective time value of the mock control. Raw data were analysed using PEAKS Studio 8.5 (Bioinformatics Solutions, Waterloo, Ontario, Canada [47]) against the combined human and HHV1 uniprot.org database (March 2017, 71,014 entries). Oxidation of methionine and carbamidomethylation of cysteines were set as dynamic and static modifications, respectively. Proteins included in the data interpretation exhibited a false discovery rate of <1%. Mass spectrometric analysis was performed at the Institute of Biochemistry, Friedrich-Alexander-Universität Erlangen-Nürnberg.

### RNA isolation, cDNA synthesis and quantitative real-time PCR (qRT-PCR) analyses

RNA was isolated from cell pellets, which were previously harvested by centrifugation and washed once with ice-cold PBS. For expression analysis of STAT3, total RNA was isolated using the QIAshredder kit (Qiagen, Germany) and the RNeasy Plus Mini kit (Qiagen) according to the manufacturer’s instructions. Reverse transcription into cDNA was performed using 0.5 µg RNA in a total volume of 20 µl, Oligo-dT primers and First Strand cDNA Synthesis kit (Invitrogen Thermo Fisher Scientific, Germany). The quantitative real-time PCR (qRT-PCR) against STAT3 and S14 was performed using 10 µl of SYBR Green qPCR 2× Mix (Biozym, Germany), 5 µl cDNA (concentration of 2.5 ng µl^−1^), 0.8 µl of sense primer (10 µM), 0.8 µl of antisense primer (10 µM) and 3.4 µl H_2_O. We used the following qRT-PCR primers: STAT3 sense (5′-AGT ATA GCC GCT TCC TGC AAG-3′), STAT3 antisense (5′-GCG TGA TTC TTC CCA CAG GCA-3′), reference transcripts S14 sense (5′-GGC AGA CCG AGA TGA ATC CTC A-3′) and S14 antisense (5′-CAG GTC CAG GGG TCT TGG TCC-3′). The qRT-PCR programme parameters were as follows: all samples were heated up to 95 °C for 3 min. Subsequently, 45 cycles of the following three steps were conducted: 15 s at 95 °C, 15 s at 61 °C and 15 s at 72 °C. Afterwards, a melting-curve analysis was performed by subjecting the samples to a temperature ramp (from 65 °C to 95 °C at 0.1 °C/s). qRT-PCRs were performed in a Touch Thermal Cycler CFX96 real-time system (Bio-Rad, CA, USA). Results were analysed in CFX Manager 3.0 software (Bio-Rad) and evaluated by normalization to the 40S ribosomal protein S14 reference gene (ΔCq) and the mock control (ΔΔCq). The final evaluation of 2^-ΔΔCq^ values was performed using Prism 10 (GraphPad).

The RT-PCR against GAPDH and gB was performed in a total volume of 20 µl with the following components: 275 ng of synthetized cDNA, 2 µl 10× Taq buffer, 0.5 µl deoxynucleoside triphosphates (concentration of 10 nmol µl^−1^), 0.3 µl Taq DNA polymerase (activity 1 U µl^−1^), 0.6 µl MgCl_2_ (concentration 25 mM), 1 µl of sense primer (concentration 10 pmol µl^−1^), 1 µl of antisense primer (concentration 10 pmol µl^−1^) and H_2_O. Primer for GAPDH sense (5′-CAC CAC CAT GGA GAA GGC TGG-3′), GAPDH antisense (5′-GAA GTC AGA GGA GAC CAC CTG-3′), gB sense (5′-CAT GCC AAG TAT TGG ACT GGA GGA G-3′), gB antisense (5′-CAC AGG TGT GTC GCC ATC GCA C-3′). The RT-PCR programme parameters for GAPDH and gB were as follows: all samples were heated up to 95 °C for 2 min. Subsequently, 30 cycles of the following three steps were conducted: 30 s at 95 °C, 30 s at 58 °C and 45 s at 72 °C. Final amplification was performed at 72 °C for 10 min. RT-PCRs were performed in a Touch Thermal Cycler CFX96 real-time system (Bio-Rad). PCR products were mixed with 6× loading-dye buffer (Thermo Fisher Scientific, Langenselbold, Germany) and loaded onto a 1.5% agarose gel supplemented with Midori Green DNA stain (Nippon Genetics, Düren, Germany). The electrophoresis was performed at 120 V for 45 min.

### Statistical analysis

For the determination of statistical significance, data points were checked for Gaussian distribution by applying the Shapiro–Wilk test. In case of normally distributed values, data were analysed using unpaired repeated measures (RM) one-way or two-way ANOVA and Dunnett’s or Tukey’s multiple comparison test. Kruskal–Wallis test (Dunn’s) was applied when values were not normally distributed. Significance was accepted for *P* values less than 0.05. **** indicates *P*≤0.0001; ***P≤0.001; **P≤0.01; *P≤0.05; and ns, not significant.

## Results

### Reduction of STAT3 in human HSV-1-infected mDCs

Based on our previous observations that HSV-1 inhibits IL-6R surface expression on mDCs [48], we here focused on a downstream acting transcription factor, STAT3. STAT3 is frequently targeted in different cell types by diverse viruses and also a known target of herpesviruses, such as KSHV and HCMV [49–51], but also HSV-1 [33]. However, it has not been investigated yet whether STAT3 is regulated in DCs upon HSV-1 infection to subvert the immune response.

**Fig. 1.**
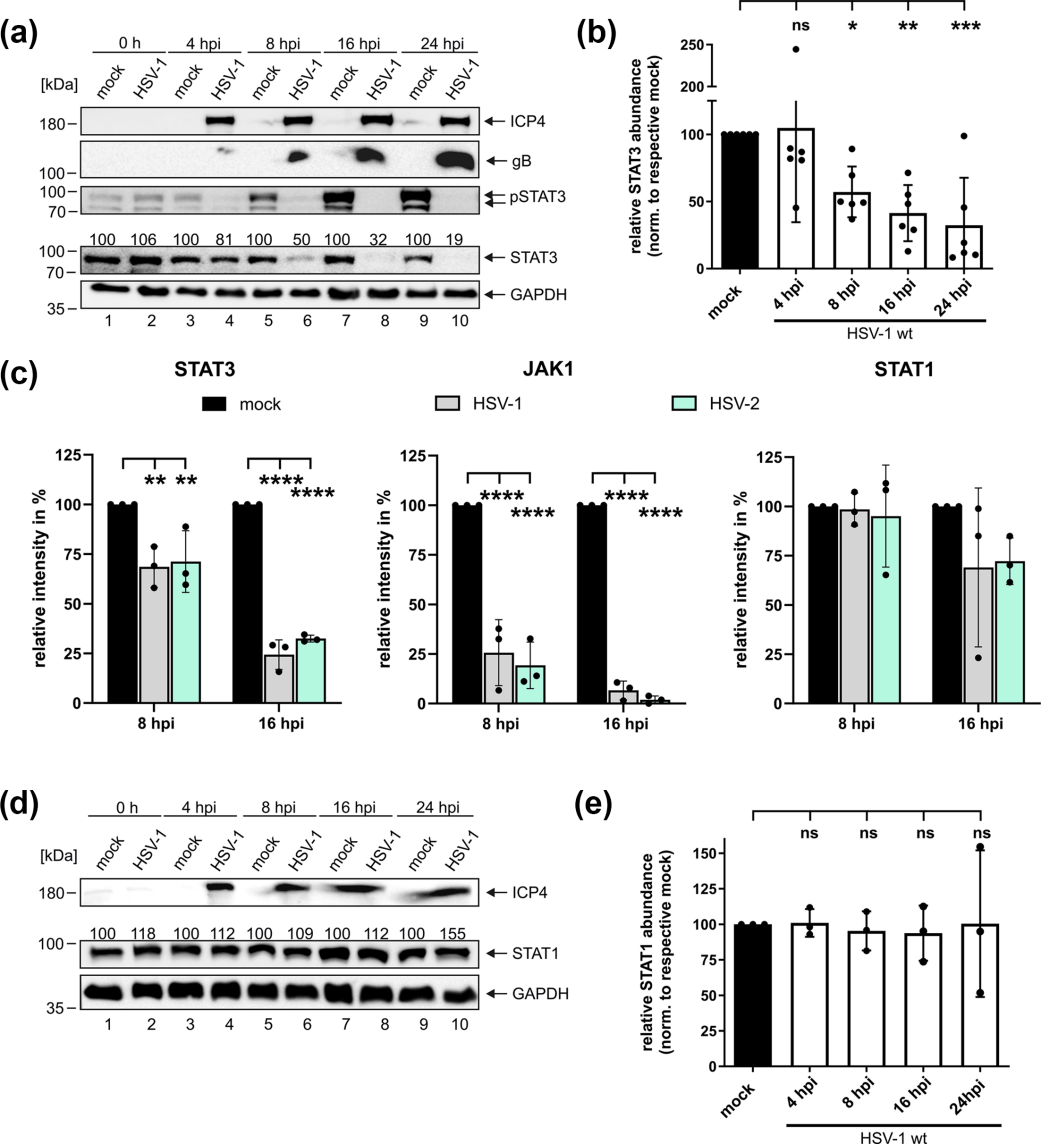
HSV-1 downregulates STAT3 and pSTAT3 protein levels in mDCs. (**a**) Mature DCs were mock- or HSV-1 wt-infected (MOI of 2) and harvested from 0 to 24 hpi. (**a**) STAT3 or (**d**) STAT1 quantification relative to GAPDH and normalized to the respective mock control is shown above the STAT3/STAT1 blot. One representative Western blot out of at least three is shown. (**b**) STAT3 abundance of six independent Western blots in (**a**) was quantified. STAT3 abundance was normalized to GAPDH and to the respective mock control. (**c**) Mature DCs were mock-, HSV-1 wt- (MOI of 2) or HSV-2-infected (MOI of 5) and harvested 8 and 16 hpi. Protein samples were used for MS-based label-free analysis of STAT3, JAK1 and STAT1. Intensities of the peptides were normalized to the mock control. Only significant changes are indicated by asterisks. (**d**) STAT1 protein expression as described in (a). (**e**) Quantification of Western blots of (d). Protein abundance of STAT1 in three independent experiments. The intensity of STAT proteins was normalized to the intensity of GAPDH and normalized to the respective mock control. Significant changes were analysed by performing (**b**) Kruskal–Wallis and Dunn’s multiple comparison test, (**c**) two-way ANOVA multiple comparison test (Tukey) or (**e**) ordinary one-way ANOVA and Dunnett’s multiple comparison test, which are indicated by asterisks (*P≤0.05, **P≤0.01, ***P≤0.001 and ****P≤0.0001). ns, not significant (*P*>0.05). Error bars indicate ±sd.

In a first step, STAT3 protein levels were analysed in mock- or HSV-1 wt-infected mDCs (MOI of 2) at different time points post-infection (0 h to 24 hpi). The 0 h time point represents the controls before the infection procedure. In the mock-treated control mDCs, we observed that the phosphorylation at tyrosine 705 of STAT3 (pSTAT3) was highly induced over time, while total STAT3 protein was equally expressed (Fig. 1a). The high phosphorylation status of STAT3 might be due to the production of IL-6 by mDCs and an autocrine feedback loop [52, 53]. Importantly, we found a strong reduction of total STAT3 protein and pSTAT3 in HSV-1-infected mDCs (Fig. 1a, lanes 4, 6, 8 and 10). The complete replication of HSV-1 in mDCs was verified by the expression of the IE and L protein ICP4 and gB, respectively (Fig. 1a). The reduction of pSTAT3 was visible as early as 4 hpi in HSV-1-infected mDCs (Fig. 1a, lane 4), while the decrease of total STAT3 protein levels was evident from eight hpi onwards, with residual protein levels of only 40% in HSV-1- versus mock-treated mDCs at 24 hpi (Fig. 1b).

We validated our results by using label-free MS and further included HSV-2 into our analysis (Table S1, available in the online Supplementary Material). By comparing the abundance of STAT3 in HSV-1-infected versus mock-treated mDCs, we confirmed a substantial reduction of STAT3 upon HSV-1 infection at 8 and 16 hpi (Fig. 1c, left graph, grey bar). Similar dysregulation of STAT3 was found in HSV-2-infected mDCs (Fig. 1c, left graph, light green bar). Both HSV-1 and HSV-2 triggered a reduction of STAT3 protein levels by 30% and 75% at 8 and 16 hpi, respectively. Interestingly, another IL-6 downstream molecule, the JAK protein, which is responsible for the phosphorylation of the intracellular domain of gp130 [22], was significantly reduced in HSV-1- and HSV-2-infected mDCs compared to the mock control (Fig. 1c, middle graph). In the next step, we investigated the specificity of STAT3 downregulation upon HSV-1 infection by monitoring the expression of another member of the same protein family, STAT1. Our MS data set revealed the presence of similar amounts of STAT1 in HSV-1-, HSV-2- and mock-infected mDCs (Fig. 1c, right graph). By quantification of STAT1 protein expression of three independent Western blots, we confirmed the results showing that STAT1 protein expression was unaffected in HSV-1-infected mDCs (Fig. 1d, e). Thus, STAT3 seems to represent a specific new target of HSV-1 to be downregulated in human mDCs.

**Fig. 2.**
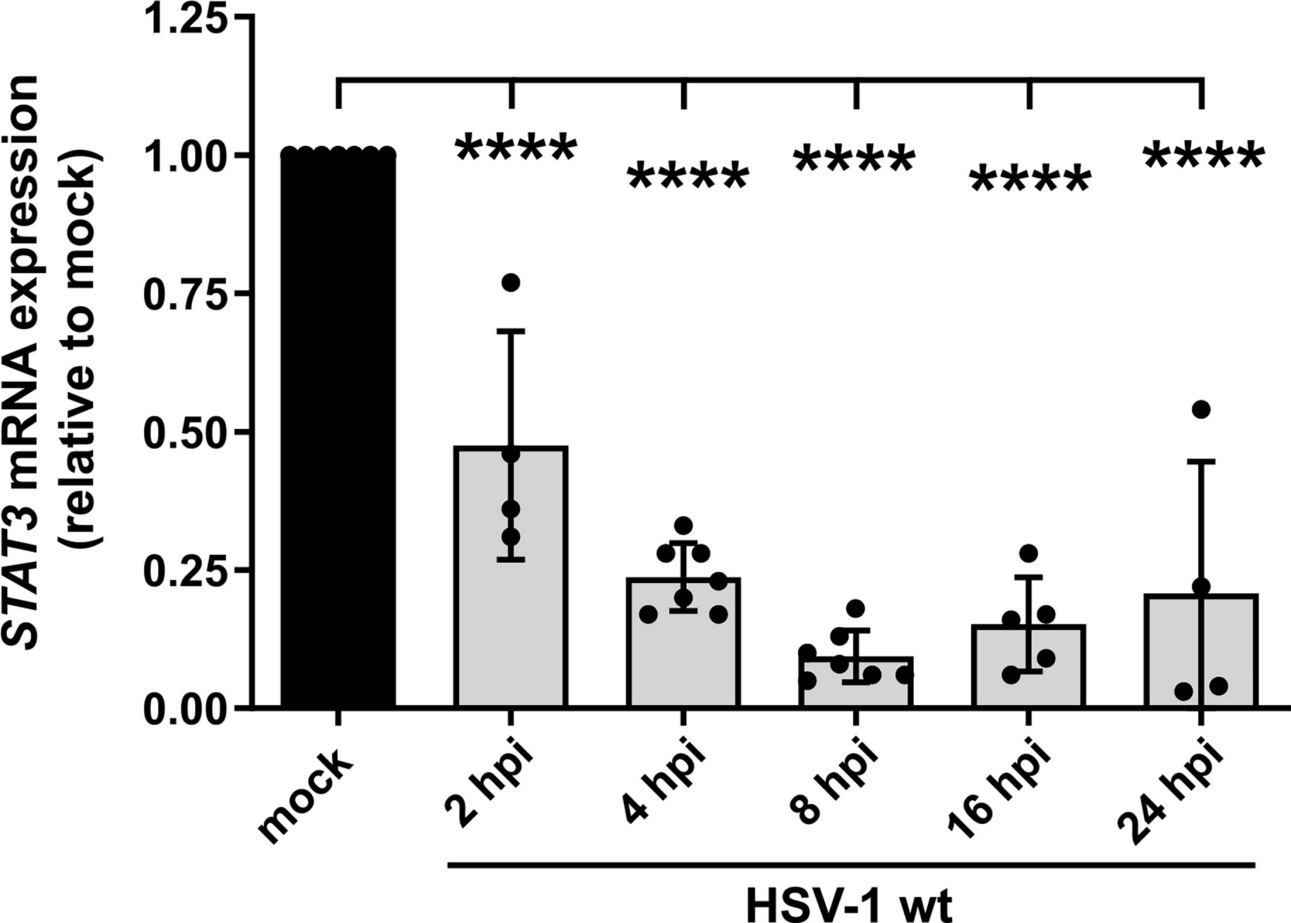
HSV-1 also reduces *STAT3* mRNA expression. Mock- and HSV-1 wt-infected (MOI of 2) mDCs were harvested from 2 to 24 hpi. RNA was subjected to qRT-PCR analysis. The expression of *STAT3* mRNA is depicted relative to the mock control and normalized to the *S14* reference gene (∆∆Cq values). Data represent independent experiments with samples from at least four different healthy donors. Data were analysed by performing ordinary one-way ANOVA and Dunnett’s multiple comparison test. *P* values <0.05 were considered significant and are indicated by asterisks (****P≤0.0001). Error bars show ±sd.

Following protein expression analyses, *STAT3* mRNA expression levels were analysed in mock- and HSV-1-infected mDCs using qRT-PCR. In line with expectations, *STAT3* mRNA levels were strongly affected in HSV-1-infected mDCs, declining from 2 hpi onwards to a maximum decrease of 90% at 8 hpi compared to mock expression levels (Fig. 2, *P*<0.0001). In contrast to STAT3 protein expression, being significantly downregulated from 8 hpi onwards (Fig. 1a), *STAT3* mRNA levels were more rapidly downregulated upon HSV-1 infection. Concerning the different time points of STAT3 mRNA and STAT3 protein downregulation following HSV-1 infection, we analysed the stability of STAT3 specifically in mDCs by inhibiting transcription and translation by Act. D or CHX, respectively. Western blot analysis shows STAT3 protein is quite stable in mDCs for at least 8 h post-CHX treatment (Fig. S1). So far, our results indicate that HSV-1 downregulates *STAT3* mRNA very early upon infection (2 hpi), which subsequently might be responsible for STAT3 protein downregulation in mDCs from 8 hpi onwards.

### Involvement of the viral proteins vhs and ICP27

**Fig. 3.**
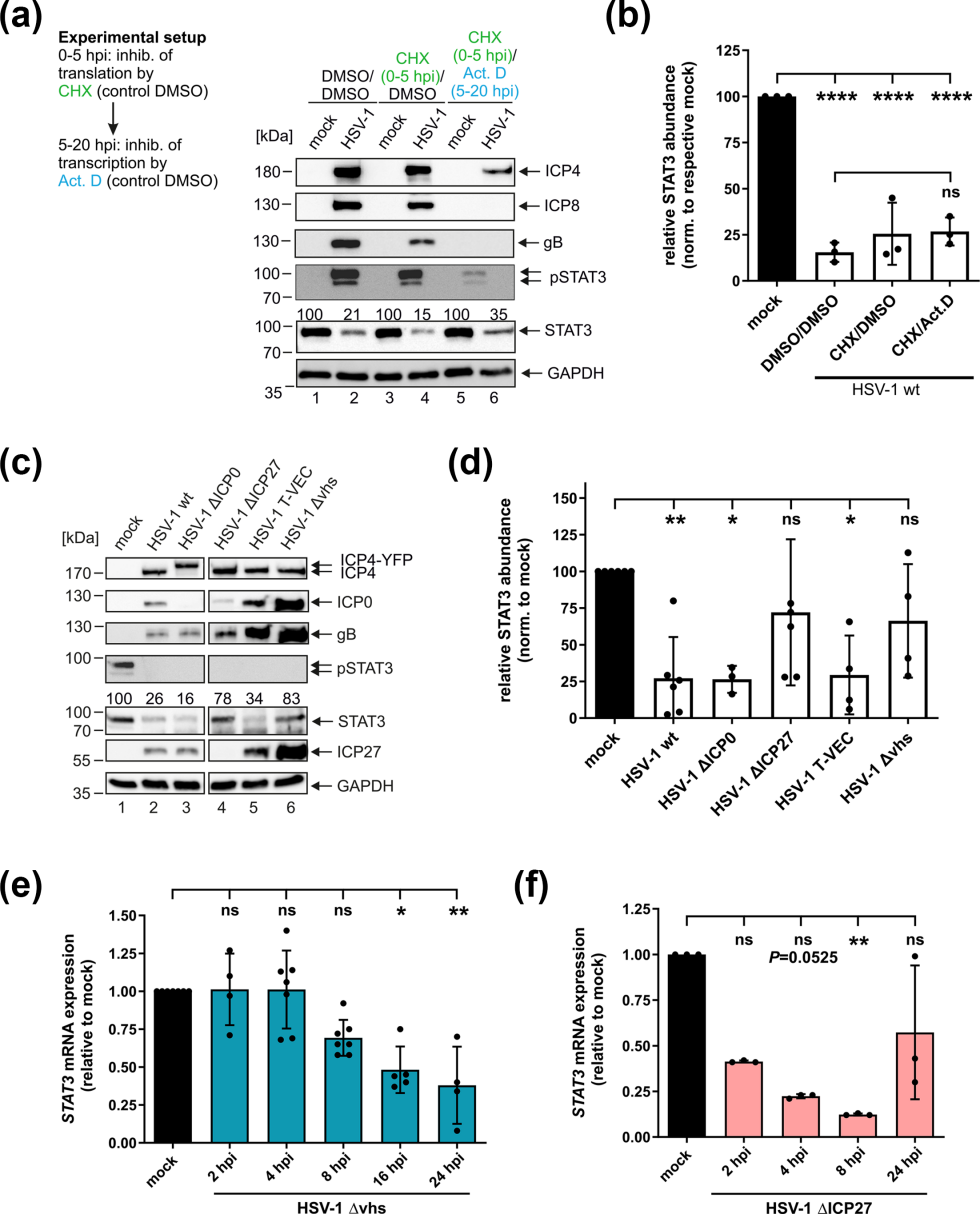
STAT3 downregulation in HSV-1-infected mDCs is dependent on vhs- and ICP27. (**a**) The experimental setup is shown on the left side. In the presence of CHX (100 µg ml^−1^), or DMSO as a control, mDCs were mock- or HSV-1 wt-infected (MOI of 2). At 5 hpi, CHX was either replaced by Act. D (5 µg ml^−1^, CHX/Act. D condition, lanes 5 and 6), or by DMSO as a control (CHX/DMSO condition, lanes 3 and 4). At 20 hpi, mDCs were harvested. STAT3 quantification is shown above the STAT3 blot. Western blots were repeated three times using cells from different healthy donors. (**b**) Quantification of STAT3 protein abundance of (a). STAT3 protein expression of three independent experiments was quantified. (**c**) Mature DCs were either mock-, HSV-1 wt-, HSV-1 ∆ICP0-, HSV-1 ∆ICP27-, HSV-1 T-VEC- or HSV-1 Δvhs-infected and harvested 24 hpi. Subsequently, protein lysates were subjected to Western blotting. STAT3 quantification relative to GAPDH and normalized to the respective mock control is shown above the STAT3 blot. Western blots were repeated three times using cells from different healthy donors. (**d**) Quantification of Western blots of (c). Protein expression of STAT3 of at least three independent experiments was quantified. The intensity of STAT3 proteins was normalized to the intensity of the reference protein GAPDH and normalized to the respective mock control. (**e, f**) Mature DCs treated as a control (mock, black bars) or infected with HSV-1 ∆vhs-infected [blue bars, MOI of 2 (**e**)] or HSV-1 ∆ICP27 [pink bars, MOI of 2 (**f**)] were harvested 2 to 24 hpi. Transcribed cDNA was subjected to qRT-PCR analyses. The expression of *STAT3* mRNA is depicted relative to the mock control and normalized to the *S14* reference gene (black bar, set to 100%, ∆∆Cq values). Significant changes were analysed by performing (**b, d**) ordinary one-way ANOVA and Tukey’s multiple comparison test or (**e, f**) Kruskal–Wallis test and Dunn’s test. *P* values <0.05 were considered significant and are indicated by asterisks (*P≤0.05, **P≤0.01, ***P≤0.001 and ****P≤0.0001). ‘ns’ indicates not significant (*P*>0.05). Error bars indicate ±sd.

In a next experiment, we sought to narrow down which subfraction of viral proteins is responsible for STAT3 downregulation. Thus, CHX–Act. D chase experiments were conducted to analyse the role of viral IE gene products. By infecting mDCs with HSV-1 wt in the presence of CHX until 5 hpi, only transcription of *IE* mRNA is processed. By replacing CHX with Act. D from 5 hpi onwards, the translation of mRNAs from the IE phase can occur; however, transcription from E and L gene phases should be blocked. This means that only incoming tegument proteins are present and a limited amount of IE gene products will be translated. The conditions DMSO/DMSO and CHX/DMSO represent the controls of the experiment. The successful inhibition of E and L gene expression following CHX–Act. D treatment was verified at transcript levels using RT-PCR against *gB* (L gene product, Fig. S2) and at protein levels by Western blotting against ICP8 (E gene product, Fig. 3a). Since pSTAT3 and STAT3 expression was similar in DMSO/DMSO and CHX/DMSO-treated mock mDCs, treatment of mDCs with CHX did not affect STAT3 expression per se (Fig. 3a, lanes 1 and 3). However, both pSTAT3 and STAT3 were downregulated upon HSV-1 infection in the presence of CHX (CHX/DMSO). Importantly, pSTAT3 and STAT3 were still strongly reduced in HSV-1-infected mDCs upon CHX/Act. D treatment, supporting the notion that IE gene products or viral tegument proteins of incoming virions mediate pSTAT3 and STAT3 downregulation (CHX/Act. D). Moreover, quantification of STAT3 protein expression in the CHX/Act. D condition revealed a significant downregulation to a similar extent compared to HSV-1-infected DMSO-treated mDCs (Fig. 3a, lanes 2, 4 and 6, Fig. 3b). In summary, these results indicate that an IE viral protein or an incoming tegument protein is critically involved in HSV-1-triggered pSTAT3 and STAT3 downregulation.

These results propose that a viral protein operating at the first stage of HSV-1 infection might be responsible for the observed STAT3 downregulation. Based on that, we now used different HSV-1 deletion strains to test the impact of a tegument protein and three out of five IE proteins on STAT3 expression. The HSV-1 T-VEC strain lacks the ICP34.5 and the IE protein ICP47, which blocks the transporter associated with antigen presentation (TAP) to inhibit the peptide transport for antigen presentation [54]. This strain originates from the virus strain T-VEC (IMLYGIC®) used as a drug for melanoma therapy [55]. While the IE protein ICP27, ablated in HSV-1 ∆ICP27, functions on mRNA export and mRNA 3′ processing [56, 57], HSV-1 ∆vhs is ablated in the viral tegument protein vhs, acting as endoribonuclease for destabilizing viral and host mRNA [58, 59]. In addition, we analysed STAT3 protein expression upon HSV-1 infection in the absence of the IE protein ICP0 (HSV-1 ∆ICP0/ICP4-YFP). The viral E3 ubiquitin ligase ICP0 acts on viral gene expression and blocks viral DNA silencing [60]. The complete replication of the different used HSV-1 deletion strains was verified by the expression of the IE and L protein ICP4 and gB, respectively (Fig. 3c). While ICP47 does not seem to be incorporated into the virions, ICP0, ICP27 and vhs are present in HSV-1 mature virions and immediately released into the host cell upon infection [44, 61, 62]. Infection of mDCs with these deletion strains revealed that STAT3 protein levels were almost completely lost upon HSV-1 wt, HSV-1 T-VEC and HSV-1 ΔICP0 infection (Fig. 3c, lanes 2, 3 and 5). In sharp contrast, STAT3 protein was substantially restored in both HSV-1 ∆vhs- and HSV-1 ∆ICP27-infected mDCs, albeit STAT3 protein levels were decreased compared to mock mDCs (Fig. 3c, lanes 4 and 6). The quantification of STAT3 protein expression confirmed these findings and revealed no significant changes in STAT3 upon infection of mDCs with ICP27 and vhs deletion strains compared to mock mDCs, but significant downregulation of STAT3 upon infection with ICP0 and ICP47 mutants (Fig. 3d). This suggests that vhs and ICP27, but not ICP0 and ICP47, could contribute to STAT3 degradation. In comparison to the total STAT3 protein levels, pSTAT3 levels were strongly downregulated upon infection with all tested deletion strains, similar to that found for HSV-1 wt (Fig. 3c, pSTAT3). Taken together, vhs and ICP27 seem to be involved in total STAT3 downregulation; however, none of the analysed viral proteins are responsible for the downregulation of pSTAT3. Thus, STAT3 and pSTAT3 are regulated differentially in mDCs.

ICP27 is important for the modulation of the host’s transcriptional machinery, and vhs mainly functions on the posttranscriptional degradation of host mRNA; however, vhs also helps to evade the host’s immune response [59, 63]. Thus, we focused in the next experiments on the regulation of *STAT3* mRNA levels upon infection with either of these two deletion strains, side-by-side with the HSV-1 wt strain as a control. In comparison to HSV-1 wt (Fig. 2), the lack of the viral tegument protein vhs caused a time-delayed reduction of *STAT3* mRNA expression levels, becoming significant at 16 hpi (Fig. 3e). In contrast, in HSV-1 ∆ICP27-infected mDCs, *STAT3* mRNA consistently decreased across experiments at 4 hpi compared to mock (*P*=0.0525), reaching statistical significance at 8 hpi (Fig. 3f). Moreover, kinetics of *STAT3* mRNA downregulation in ∆ICP27-infected mDCs were comparable to those of HSV-1 wt-infected mDCs from 2 to 8 hpi (see Fig. 2). In summary, while HSV-1 vhs and ICP27 are involved in total STAT3 protein downregulation (Fig. 3c), only vhs also affects *STAT3* transcript levels upon infection.

### Proteasome-dependent degradation of STAT3 in HSV-1-infected mDCs

**Fig. 4.**
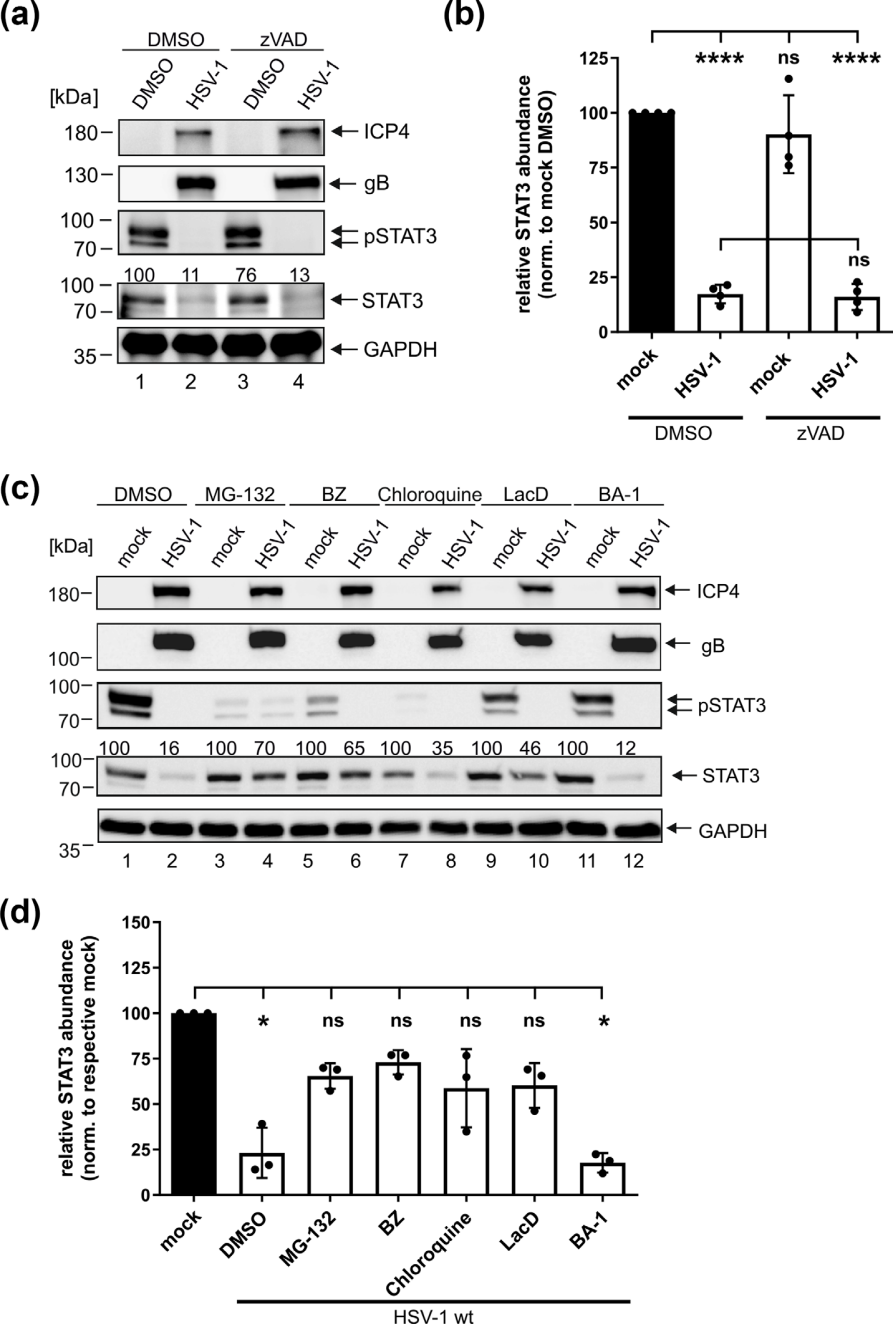
STAT3 is degraded by an apoptosis-independent but proteasome-dependent mechanism. (**a**) At 30 min prior to the infection, mDCs were treated with the pan-caspase inhibitor zVAD (20 µM) or DMSO as a control. Afterwards, mDCs were mock- or HSV-1 wt-infected in the presence of DMSO or zVAD. One representative Western blot out of four is shown. (**a, c**) Cells were harvested 16 hpi (**a**) or 24 hpi (**c**), and complete lysates were subjected to Western blotting. STAT3 quantification relative to GAPDH and normalized to the mock DMSO control is shown above the STAT3 blot. (**b**) Quantification of Western blots in (a). Protein abundance of STAT3 in three independent experiments was quantified. (**c**) Mature DCs were mock- and HSV-1 wt-infected (MOI of 2) and treated 1 hpi with different proteasome inhibitors: MG-132 (10 µM), BZ (2 µM), LacD (10 µM), BA-1 (500 mM) or chloroquine (50 µM). One representative Western blot out of three is shown. (**d**) Quantification of Western blots in (c). Quantification of STAT3 protein expression in at least three independent experiments. The intensity of STAT3 was normalized to GAPDH and to the respective mock control. Significant changes were analysed by performing an (**b**) ordinary one-way ANOVA and Tukey’s multiple comparison test or (**d**) Kruskal–Wallis test and Dunn’s test. *P* values <0.05 were considered significant and are indicated by asterisks (*P≤0.05, ****P≤0.0001). ‘ns’ indicates not significant (*P*>0.05). Error bars indicate ±sd.

We demonstrated that STAT3 is downregulated at the protein and mRNA levels upon HSV-1 infection in mDCs. However, upon infection, HSV-1 also induces apoptosis in iDCs and other cell types, such as monocytes [11, 64, 65]. Since other viruses, such as vaccinia virus and measles virus, but also the herein tested proteasome inhibitors, lead to apoptosis in mDCs, we analysed whether STAT3 downregulation is additionally induced by apoptosis [66, 67]. To do so, mDCs were mock- or HSV-1 wt-infected in the presence of the pan-caspase inhibitor zVAD, or DMSO as a control. The inhibition of apoptosis using 20 µM zVAD was checked by staining against Annexin (APC) and 7AAD (PE-Cy5) in mock- and HSV-1-infected mDCs of each donor, where 7AAD-negative and Annexin-positive cells represent the apoptotic cells (Fig. S3A). The effect of zVAD on STAT3 protein levels was monitored at 16 hpi. Despite the application of the pan-caspase inhibitor zVAD, both pSTAT3 and STAT3 were still drastically downregulated in HSV-1-infected mDCs (Fig. 4a, lane 4). Moreover, upon HSV-1 infection, pSTAT3 and STAT3 protein amount in zVAD-treated mDCs was as abundant as in DMSO-treated mDCs and not significantly changed (Fig. 4b). In conclusion, pSTAT3 and STAT3 downregulation in mDCs is not a result of HSV-1-mediated apoptosis.

Based on our results at the protein levels, the STAT3 protein seems to be dependent on an additional mechanism, apart from the decrease in *STAT3* mRNA. To test this, inhibitors targeting either the proteasome or autophagy were used upon HSV-1 infection. For blocking the proteasome, MG-132, BZ and LacD were used after infection [68, 69], while chloroquine and BA-1 are both inhibitors of autophagy and lysosomal degradation, which were applied prior to infection [70, 71]. Interestingly, pSTAT3 protein levels in mock-treated cells were completely lost in mDCs treated with MG-132 and chloroquine (Fig. 4c, lanes 3 and 7), while pSTAT3 was affected to a lesser extent in BZ-, LacD- and BA-1-treated mock conditions (Fig. 4c, lanes 5, 9 and 11). This suggests that phosphorylation of STAT3 is dependent on mechanisms associated with the proteasomal and autophagosomal pathways per se. By blocking the proteasome using MG-132, BZ or LacD upon HSV-1 infection, STAT3 protein levels were substantially restored compared to the HSV-1 wt-infected DMSO control (Fig. 4c, lanes 2, 4, 6 and 10). Moreover, the quantification of STAT3 protein expression shows that MG-132, BZ, LacD and chloroquine revert HSV-1-induced STAT3 downregulation, showing no significant change compared to the mock control (Fig. 4d). However, treatment with chloroquine already affected STAT3 protein expression in the mock control. In contrast, treatment with BA-1 did not prevent STAT3 downregulation in mDCs upon HSV-1 infection (Fig. 4c, lanes 8 and 12). Thus, we demonstrate that STAT3 is degraded via a proteasomal, but not autophagosomal mechanism upon HSV-1 infection in mDCs. Since pSTAT3 protein levels were reduced both in the HSV-1 wt-infected DMSO control and in the inhibitor-treated HSV-1 wt-infected mDCs, pSTAT3 protein levels are not downregulated by any of the two analysed pathways upon HSV-1 infection. Taken together, HSV-1 seems to regulate STAT3 protein at multiple levels: (1) by downregulation of *STAT3* mRNA early after infection, (2) via the function of vhs and ICP27 and (3) additionally via the proteasome, but independent of the viral E3 ubiquitin ligase ICP0.

### STAT3 dysregulation using HSV-1 clinical isolates

**Fig. 5.**
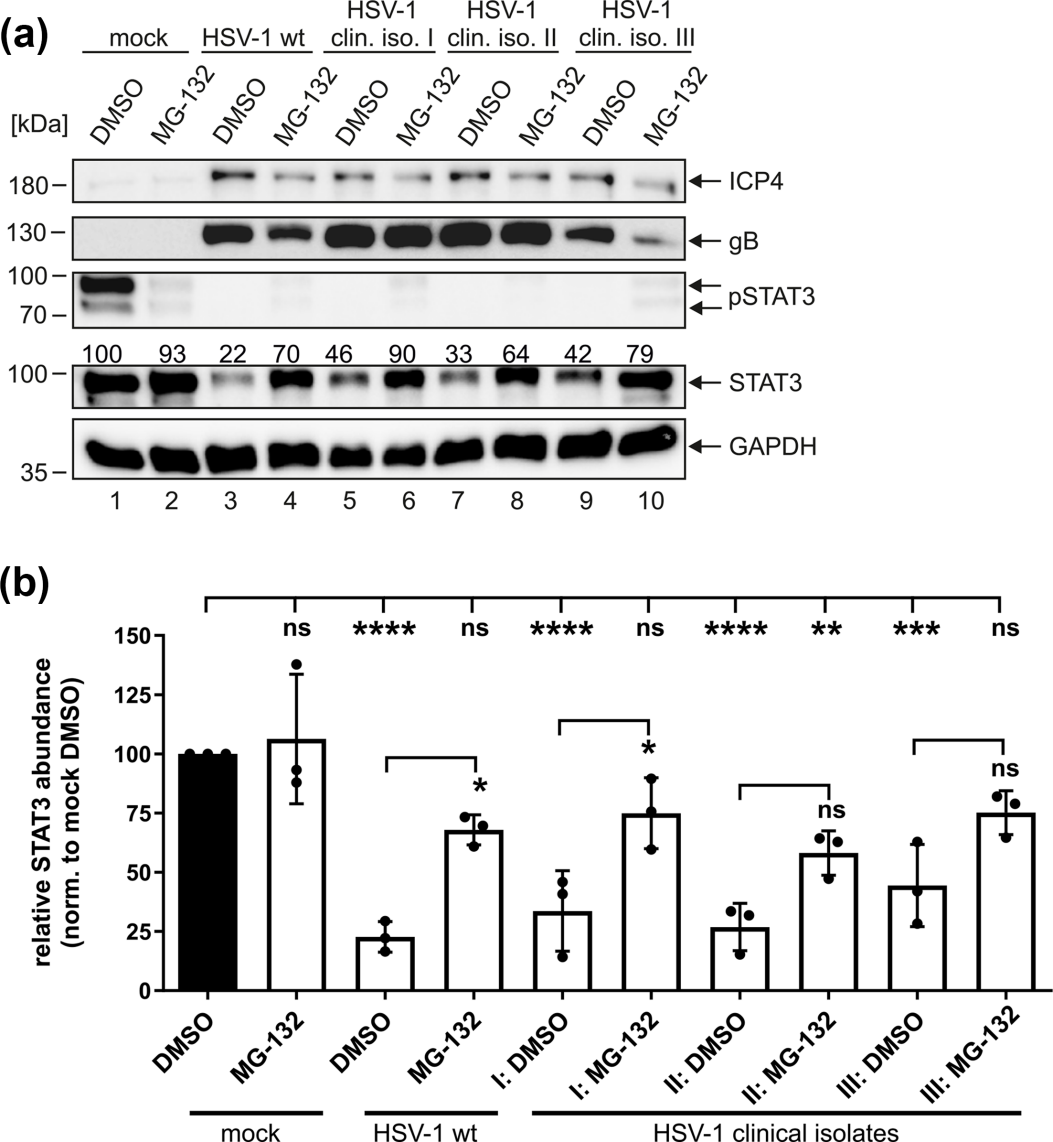
HSV-1 clinical isolates degrade STAT3 in a proteasome-dependent manner. (**a, b**) Mature DCs were treated as a control (mock), infected with HSV-1 wt (MOI of 2) or with HSV-1 strains isolated from patients (clin. iso., MOI of 2). At 1 hpi, all conditions were either treated with the proeasome inhbitor MG-132 (10 µM) or DMSO. Cells were harvested 16 hpi, and cell lysates were subjected to Western blotting. (**a**) One representative Western blot out of three is depicted. STAT3 quantification relative to GAPDH is shown above the STAT3 blot. (**b**) STAT3 protein abundance was quantified in at least three independent experiments. The intensity of STAT3 was normalized to GAPDH and to the mock DMSO control. Significant changes were analysed by performing an RM one-way ANOVA and Tukey’s multiple comparison test. *P* values <0.05 were considered significant and are indicated by asterisks (*P≤0.05, **P≤0.01, ***P≤0.001 and ****P≤0.0001). ns, not significant (*P*>0.05). Error bars indicate ±sd.

In previous experiments, we used a modified HSV-1 wt strain. To further analyse whether the observed modulation of pSTAT3 and STAT3 expression is unique to the herein used modified laboratory HSV-1 strain, we infected mDCs for 16 h with HSV-1 strains isolated from patients suffering from acute HSV-1 infections. Additionally, we used MG-132 to validate the proteasome dependence of STAT3 downregulation. Again, we monitored complete clearance of pSTAT3 in mock mDCs in the presence of MG-132 (Fig. 5a). As observed upon HSV-1 wt infection, STAT3 protein levels were significantly reduced in mDCs infected for 16 h with three different clinical isolates (Fig. 5a, lanes 5, 7 and 9). More interestingly, using the proteasome inhibitor MG-132, STAT3 protein degradation was equally prevented upon infection with HSV-1 wt and with HSV-1 clinical isolates I and III (Fig. 5a, lanes 6 and 10). Treatment with MG-132 upon infection with clinical isolate II still led to significant downregulation of STAT3 protein abundance; however, compared to the infected DMSO control, STAT3 protein expression was restored in all clinical isolates to varying degrees (Fig. 5b). Quantification of STAT3 protein expression underlines the significant downregulation of STAT3 upon infection using clinical isolates (Fig. 5b). Thus, our data show that clinical isolates of HSV-1 also trigger proteasomal degradation of STAT3 protein in mDCs.

## Discussion

HSV-1 functionally changes DCs to circumvent the immune response by targeting different cellular molecules, such as CD83, CYTIP or IL6-R [16, 44, 48, 72, 73]. Here, we identified STAT3 to be regulated at mRNA, protein and phosphorylation levels in DCs. We present how HSV-1 shapes total protein expression and phosphorylation levels of STAT3 in human mDCs. Our data provide evidence that HSV-1 affects STAT3 protein levels by targeting *STAT3* mRNA at an early stage of infection via the viral proteins vhs and ICP27 and the proteasome machinery.

STAT3 represents the downstream molecule of the mitogen-activated protein kinase and the JAK pathway [20, 21]. By binding to the receptor complex gp130-IL-6R and the induced IL-6 signalling, STAT3 is phosphorylated and translocated as a STAT3 dimer into the nucleus. Besides regulating proliferation, survival, differentiation and apoptosis [25–27], STAT3 especially plays an important role in DC function and has antiviral features, which are frequently counteracted by several viruses [28, 29, 33, 74]. Based on our previous report, showing that HSV-1 modulates IL-6R expression on HSV-1-infected mDCs [48], we here focused on the downstream acting transcription factor STAT3.

By unbiased MS and targeted Western blot analysis, we discovered STAT3 as a new HSV-1-specific target in mDCs and, additionally, its regulation by HSV-2. We demonstrate the downregulation of STAT3 and pSTAT3 protein levels in HSV-1 wt-infected mDCs compared to the mock control (Fig. 1a–c). Years of intensive research revealed that modulation of STAT3 strongly depends on the cell type and virus under investigation, which has yet to be analysed in human mDCs [31, 32]. In comparison to STAT3 regulation in HSV-1-infected mDCs, the beta herpesvirus HCMV interferes with STAT2 phosphorylation in human MRC-5 fibroblasts [75]. Moreover, HCMV triggers shuttling of unphosphorylated STAT3 into the nucleus to foster viral DNA replication in MRC-5 cells fibroblasts, suggesting an antiviral role of STAT3 [51]. The γ-herpesvirus EBV reduces STAT3 protein levels reflected by the low expression of STAT3 in the EBV-positive Hodgkin lymphoma cell line AM-HLH [76]. The viral protein HBx of the hepatitis B virus promotes pSTAT3 dimerization to enhance viral replication in HepG2.2.15 cells [77, 78]. Especially for DCs, STAT3 was described to affect maturation. STAT3 knock-down in mDCs results in increased phosphorylation of other STAT molecules, e.g. STAT1 and STAT5 [29]. Thus, the increase of pSTAT3 during the cultivation of mock-infected mDCs might be a result of previous maturation. Additionally, it is known that IL-6 induces an autocrine feedback loop by binding of the cytokine to the respective receptor, IL-6R. Since IL-6 is produced by mDCs, the induced pSTAT3 protein levels might be owing to the produced IL-6 and the autocrine IL-6 effect [52, 79]. During the last years of research, it became clear that latency and reactivation might be regulated by DCs, meaning that DCs maintain HSV-1 latency in trigeminal ganglia [80]. In mice, the subtype CD8*α*^+^ DCs seem to be involved in latency, in particular higher amounts of this DC subtype fostering latent genome numbers in ganglia [34]. Moreover, STAT3 has been reported to have regulatory functions in regard to HSV-1 latency in ganglia. Du and colleagues blocked STAT3 by inhibitor treatment, leading to the upregulation of viral gene transcription of the alpha, beta and gamma genes and thus reactivation of HSV-1 [35]. Whether this function and role is also true in monocyte-derived DCs has to be investigated. Influenza A virus and vaccinia virus were reported to reduce STAT3 expression to antagonize the antiviral activity of STAT3, required to activate IFN*α*-mediated gene expression [81]. Based on the different regulation of STAT3 among various cell types and herpesviruses, it would be interesting whether the here observed STAT3 downregulation in DCs is a conserved mechanism in *α*-, *β*- and *γ*-herpesviruses. Since DCs, in particular plasmacytoid DCs, are essential to clear virus infections by producing type I IFNs, the here shown downregulation of STAT3 in HSV-1-infected mDCs might be useful to subvert the antiviral immune response. Further supporting a possible antiviral role of STAT3 is the observation that mice, lacking STAT3, were more susceptible to HSV-1 compared to the control [33]. However, it remains uncertain whether HSV-1 also depletes STAT3 in murine DCs similarly to human DCs as presented here.

Apart from the herein identified reduced STAT3 protein levels in HSV-1 wt-infected mDCs, STAT1 protein amounts were stably expressed in DCs (Fig. 1d, e). This indicates that STAT3 is specifically targeted by HSV-1 in mDCs. Similar observations upon HSV-1 infection were reported in HeLa cells, showing STAT3 downregulation while STAT1 was stably expressed [82]. Besides the regulation of STAT3 at the protein level, HSV-1 also downregulated *STAT3* at the mRNA level (Fig. 2). Supporting our mRNA data, previously performed transcriptomic analysis in human fetal foreskin fibroblast (HFF) cells also revealed *STAT3* downregulation upon HSV-1 infection at 12 hpi [83]. In mDCs, HSV-1 regulates *STAT3* mRNA at very fast kinetics (Fig. 2). Thus, we propose that HSV-1 first regulates *STAT3* mRNA levels, which consequently results in STAT3 protein downregulation (Figs 1a and 2). Since the half-life of STAT3 protein has been described to be around 8 h in COS-7 cells [84] and we additionally checked the stability of STAT3 in mDCs (Fig. S1), it is not surprising that the mRNA regulation is initially effective on protein levels starting from 8 hpi onwards. HSV-1 specifically modulates the host’s environment to efficiently replicate and propagate. The impact on STAT3 downregulation seems to be conserved between HSV species, since MS label-free analyses showed diminished STAT3 protein amounts in HSV-2-infected samples as well (Fig. 1c). Moreover, three clinical isolates of HSV-1 led to comparable STAT3 and pSTAT3 depletion compared to the laboratory HSV-1 strain (Fig. 5).

To get more insights into the mechanism of STAT3 downregulation, we performed infection experiments in the presence of CHX and Act. D, where STAT3 protein expression was still reduced to the same extent as in the DMSO control upon HSV-1 infection (Fig. 3a). This suggests that the expression of limited amounts of IE proteins and tegument proteins, which are released immediately after cell penetration, is sufficient for STAT3 protein downregulation. The HSV-1-expressed IE proteins, i.e. ICP0, ICP4, ICP22, ICP27 and ICP47, were already reported to modulate the host’s immune response. For instance, the viral IE protein ICP47 blocks the TAP protein, which consequently prevents the peptide loading onto MHC class I molecules and the activation of CD8^+^ T cells [85]. Moreover, ICP27 was described to modulate IFN expression by interfering with the cGAS-STING-TBK1 pathway [86]. Especially relevant for DCs, the IE protein ICP0 was described to be responsible for the degradation of the surface molecule CD83 in HSV-1-infected mDCs and dysfunction of DCs [16, 73]. However, analysing STAT3 protein downregulation, the viral E3 ubiquitin ligase ICP0 seems to be dispensable for STAT3 downregulation (Fig. 3c). Here, ICP27 as an IE protein was identified to be involved in STAT3 protein downregulation (Fig. 3c). By contrast, *STAT3* mRNA was still reduced in HSV-1 ∆ICP27-infected mDCs and was comparable to the time course in HSV-1 wt-infected mDCs. Focusing on the function of the IE protein ICP27, shuttling mRNA for consequent inhibition of nuclear export and translation into proteins [63], the deletion of ICP27 might only affect protein levels of STAT3 and not the mRNA (Fig. 3c, f). Yet, we cannot exclude that the rather unaffected or only slightly affected STAT3 protein levels following infection with HSV-1 ∆ICP27 are a result of the impaired expression of vhs in this mutant [87]. Vhs is a tegument protein, which is released directly after host cell penetration and thus functions at the very beginning of HSV-1 infection. Deletion of vhs also prevented STAT3 protein downregulation upon HSV-1 infection. Further, infection with HSV-1 ∆vhs led to time-delayed *STAT3* mRNA downregulation compared to HSV-1 wt (Fig. 3c, e). Thus, vhs, encoded by the *UL41* gene and degrading cellular mRNA as an endoribonuclease, is one responsible factor for STAT3 downregulation, but not the only one [59, 88]. Transcriptomic data and mathematical models estimate the highest activity of vhs in HSV-1-infected HFF cells directly after infection, falling down to 10% of the activity of mock-infected cells at 8 hpi [89]. Moreover, Friedel and colleagues suggested that vhs degrades around 25–30% of cellular mRNA in the first 8 h of infection [89], perfectly fitting to the early *STAT3* mRNA loss in HSV-1-infected mDCs in our experiments (Fig. 2). Upon lytic infection of iDCs, HSV-1 induces apoptosis for efficient viral replication [90], mediated by the downregulation of the cellular FLICE-inhibitory protein (c-FLIP), which promotes caspase-8-dependent apoptosis [64]. Moreover, the *α*-herpesvirus VZV is described to activate STAT3 by phosphorylation for inhibition of apoptosis [91]. However, our results in mDCs exclude apoptosis as a cause of STAT3 downregulation upon HSV-1 infection (Fig. 4a, b).

Besides the regulation at the *STAT3* mRNA levels, mechanistically, our data hint towards a proteasome-dependent degradation of STAT3 in HSV-1-infected mDCs (Fig. 4c, d). Since the ICP0 deletion mutant still led to STAT3 downregulation (Fig. 3c), we speculate on a viral E3 ubiquitin-ligase independent mechanism of STAT3 degradation. Focusing on the proteasome machinery, STAT3 protein might either be ubiquitinated and degraded by the E1, E2 and E3 protein cascade or degraded in a ubiquitin-independent manner [92]. The latter mechanism is less frequently used for protein degradation compared to ubiquitin-targeting of proteins for subsequent degradation, however, more prevalent than expected in the last decades [93]. In this regard, in HSV-1-infected mDCs, a ubiquitin-independent mechanism of degradation of the surface receptor CD83 in HSV-1-infected mDCs was already reported [16]. Taken together, HSV-1 seems to regulate STAT3 protein on multiple levels. Quickly after infection, HSV-1 downregulates *STAT3* mRNA, which is followed by reduced protein levels with the contribution of the viral proteins vhs and ICP27 and protein degradation via the proteasome later upon infection. Concerning pSTAT3 protein levels, they seem to be differentially regulated independent of the mentioned viral proteins and the proteasome machinery.

Together, we show that HSV-1 substantially interferes with STAT3 protein expression and phosphorylation in mDCs. These data, together with our previous report [48] suggest that HSV-1 specifically targets several components of the IL-6 signalling pathway, i.e. IL-6R, JAK1 and STAT3, very likely to evade the host’s immune response in mDCs.

## Supplementary material

10.1099/jgv.0.002280 Fig. S1.

10.1099/jgv.0.002280 Table S1.

## Abbreviations

Act. D: actinomycin D
BA-1: bafilomycin A1
BHK: baby hamster kidney
BZ: bortezomib
CCR: CC chemokine receptor
CD: cluster of differentiation
CHX: cycloheximide
CMV: cytomegalovirus
DC: dendritic cell
E: early
GAPDH: glyceraldehyde-3-phosphate dehydrogenase
gB: glycoprotein B
GM-CSF: granulocyte-macrophage colony-stimulating factor
HCMV: human cytomegalovirus
HFF: human fetal foreskin fibroblast
hpi: hours post infection
HSV-1: herpes simplex virus type 1
ICP: infected cell protein
iDC: immature dendritic cell
IE: immediate-early
IL-6R: IL-6 receptor
JAK: Janus kinase
KSHV: Kaposi’s sarcoma-associated herpesvirus
L: late
LacD: lactacystin D
mDC: mature dendritic cell
MHC: major histocompatibility complex
pSTAT: phosphorylated signal transducer and activator of transcription
qRT-PCR: real-time quantitative PCR
RM: repeated measures
RPMI: Roswell Park Memorial Institute
STAT: signal transducer and activator of transcription
TAP: transporter associated with antigen presentation
T-VEC: talimogene laherparepvec (IMLYGIC®)
UL: unique long
vhs: virion host shutoff
VZV: Varicella Zoster virus
