# Supplementary material for: Herpes simplex virus type 1 interferes with signal transducer and activator of transcription 3 expression in infected mature dendritic cells
Source: J Gen Virol. 2026 Aug 12;107(8):002280. doi: 10.1099/jgv.0.002280 (PMC13470599; doi:10.1099/jgv.0.002280)
Supplement: Supplementary file 1 [file jgv-107-02280-s001.pdf]

# Figure S1

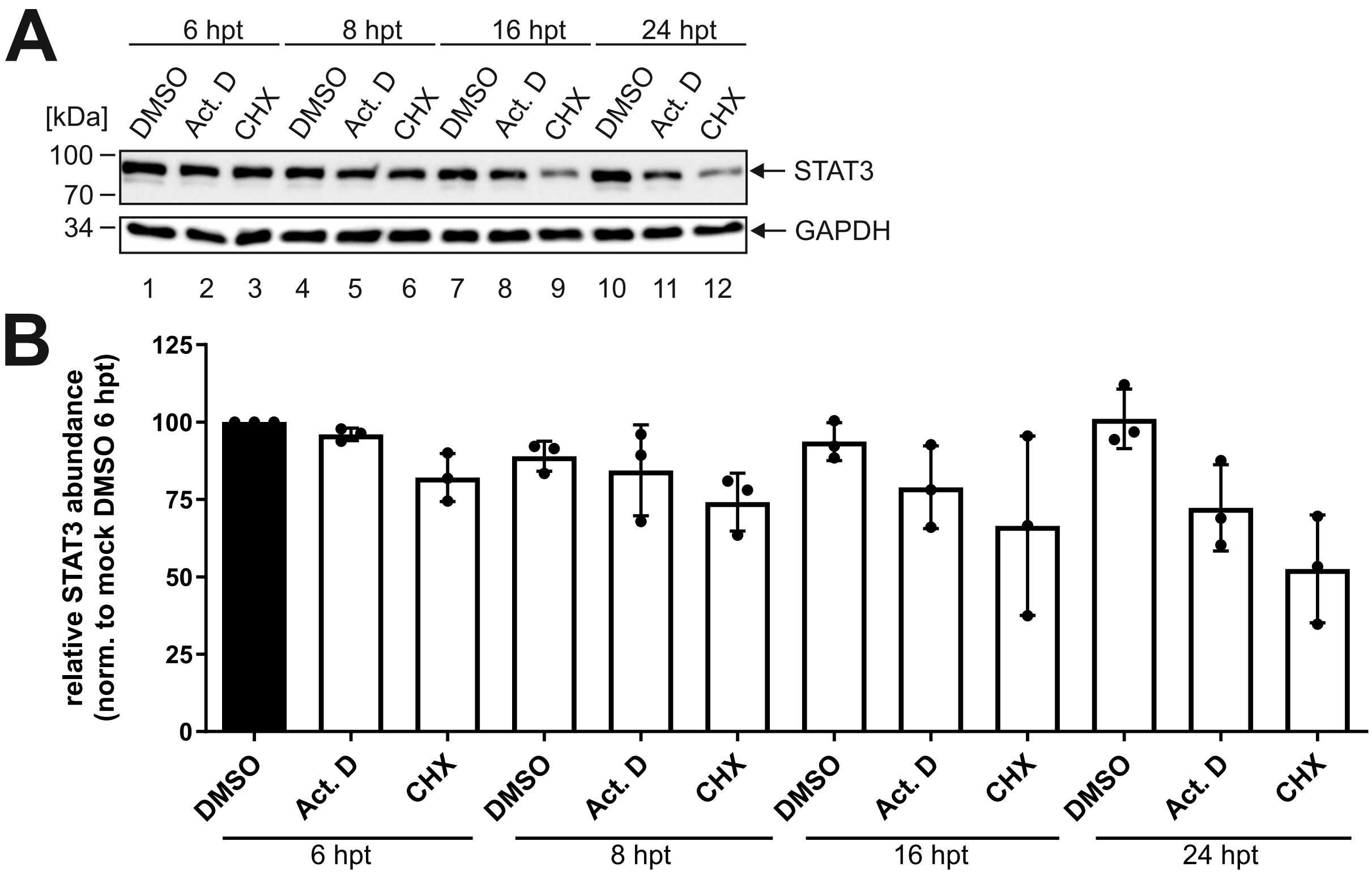


**Figure S1: STAT3 expression in the presence of Actinomycin D (Act. D) or Cycloheximid (CHX). (A, B)** Mature DCs were incubated in DC medium supplemented with either DMSO (control), Act. D (5 µg/mL) or CHX (100 µg/mL). Cells were harvested at 6 hours post treatment (hpt), 8 hpt, 16 hpt and 24 hpt. **(A)** Subsequently, protein lysates were subjected to Western blotting. One representative Western blot out of three is shown. **(B)** STAT3 protein expression of at least three independent experiments were quantified. The intensity of STAT3 was normalized to GAPDH and to mock DMSO 6 hpt. Significant changes were analyzed by performing a RM one-way ANOVA and Dunnett's multiple comparison test, revealing no significant changes (P values <0.05). Error bars indicate ± SD.

# Figure S2

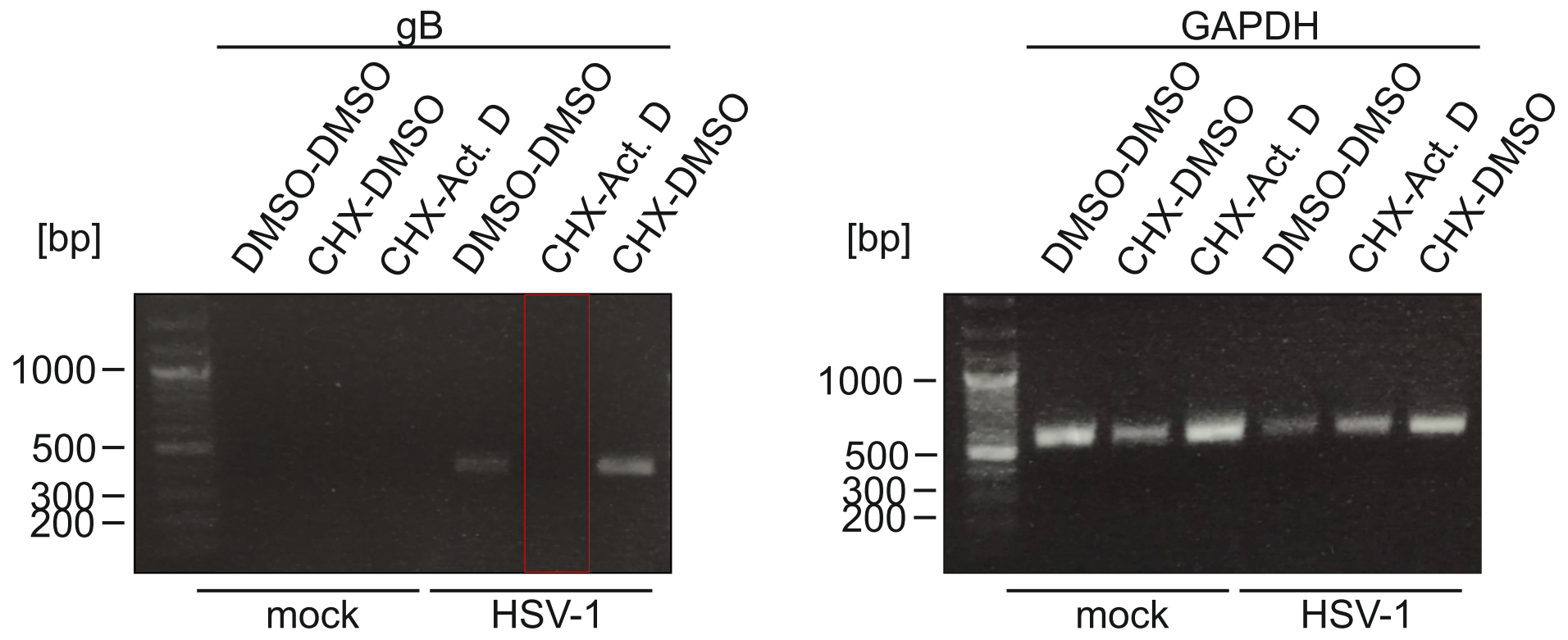


**Figure S2: Late gene expression is blocked in CHX-Act. D chase experiment.** In the presence of CHX (100 µg/mL), or DMSO as control, mDCs were mock- or HSV-1 wt-infected (MOI of 2). 5 hours later, CHX was replaced by Act. D (5 µg/mL), or DMSO as control. At 20 hpi, mDCs were harvested and isolated RNA was subjected to RT-PCR. RT-PCR samples were loaded on a 1.5 % agarose gel visualizing gB (late gene) and GAPDH (reference gene). One representative experiment out of three is depicted.

# Figure S3

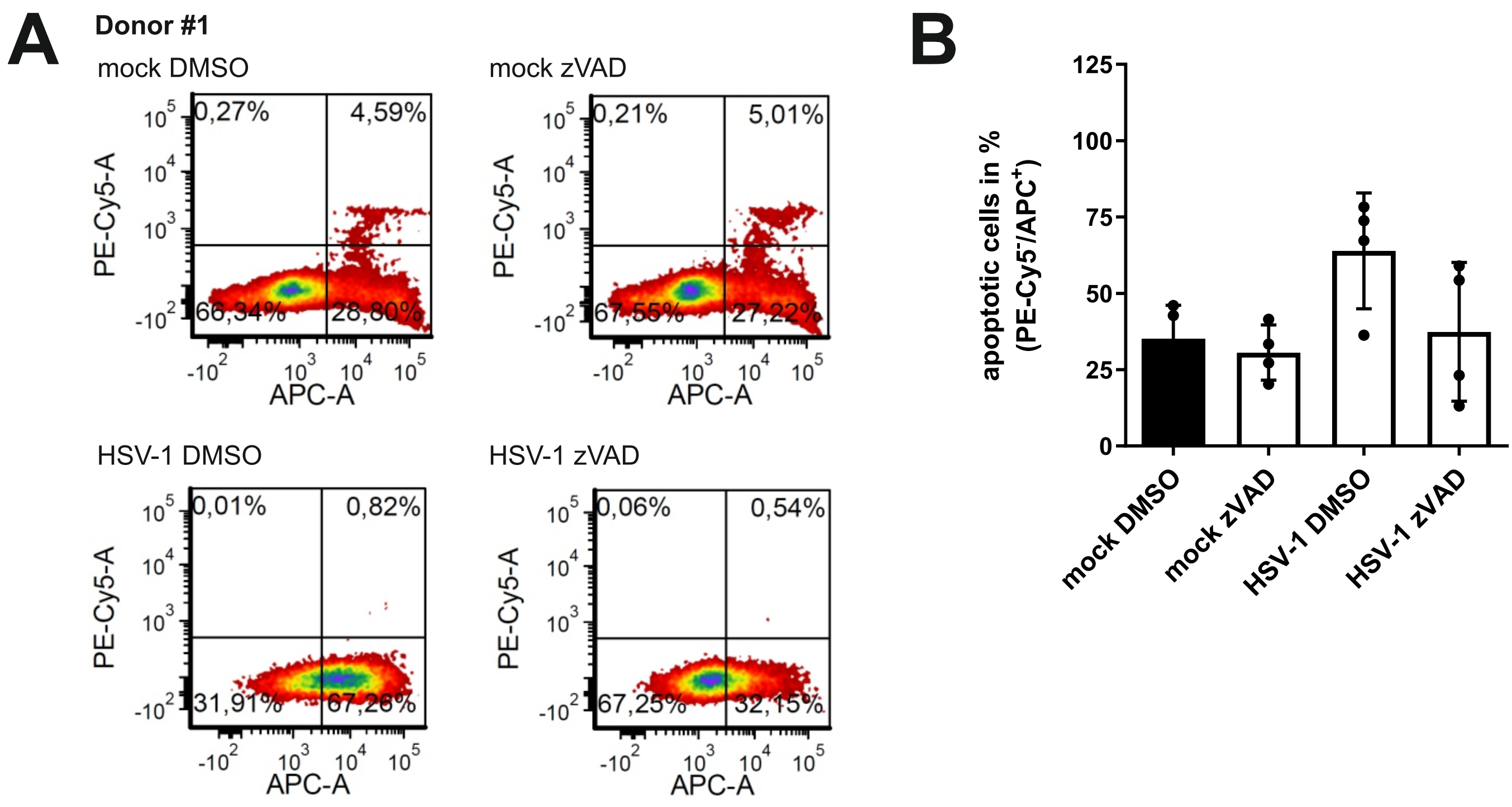


**Figure S3: Inhibition of apoptosis using the pan-caspase inhibitor zVAD. (A, B)** At 30 min prior to the infection, mDCs were treated with the pan-caspase inhibitor zVAD (20 µM) or DMSO as control. Afterwards, mDCs were mock- or HSV-1 wt-infected in the presence of DMSO or zVAD. Cells were harvested 16 hpi and stained against Annexin (APC) and 7AAD (PE-Cy5) for flow cytometry analysis. **(A)** The staining of each condition is depicted for one representative healthy donor (donor #1). **(B)** Frequency of apoptotic cells ($PE\text{-}Cy5^-/APC^+$) from flow cytometric staining of Annexin and 7AAD for all donors. Statistical analyses by performing RM one-way ANOVA and Tukey's multiple comparison test revealed no significant changes.
